# Industrial-Scale Brownmillerite Formation in Oxygen-Blown Basic Oxygen Furnace Slag: A Novel Stabilization Approach for Sustainable Utilization

**DOI:** 10.3390/ma18102182

**Published:** 2025-05-09

**Authors:** Yao-Hung Tseng, Yu-Hsien Kuo, Meng-Hsun Tsai

**Affiliations:** 1 New Materials Research and Development Department, China Steel Corporation, Kaohsiung 81233, Taiwan; 2 Intellectual Property and Testing Technology Department, China Steel Corporation, Kaohsiung 81233, Taiwan; 179259@mail.csc.com.tw (Y.-H.K.); 204362@mail.csc.com.tw (M.-H.T.)

**Keywords:** BOF slag, slag modification, brownmillerite, utilization

## Abstract

This study introduces an innovative process for stabilizing BOF slag by blowing oxygen into molten slag, addressing challenges associated with conventional methods that require silica injection. Molten BOF slag from a steelmaking workshop at China Steel Corporation is directly modified at the slag modification station, where chemical compositions and crystalline phases are analyzed under varying oxygen injection amounts. In 70 industrial trials (20–25 tons per trial) with the basicity of the BOF slag ranging from 2.2 to 4.5, the reduction in the slag expansion rate increases proportionally with oxygen-blowing amounts. Oxygen blowing facilitates the oxidation of FeO to Fe_2_O_3_, which reacts with f-CaO to form volumetrically stable C_2_AF (brownmillerite, Ca_2_Al_x_Fe_2−x_O_5_), as confirmed by XRD and SEM-EDX analyses. The treated BOF slag exhibits excellent volumetric stability (expansion < 0.5%), lower pH (10.6–10.8) in comparison to original BOF slag, and compliance with Taiwan’s EPA-leaching regulations. This stabilized slag demonstrates potential for engineering applications, such as pavement bricks, concrete products, and high-value engineered stones. Additionally, the high brownmillerite content highlights its promise for low-carbon cement applications, offering a scalable and cost-effective solution for BOF slag utilization in the steel industry.

## 1. Introduction

The applications of BOF slag are normally related to its properties, which are mainly governed by its compositions, crystalline phases, and crystallinity. Despite the fact that many post-treatments have been made to manipulate the properties of BOF slag, the changes in the compositions, crystalline phases, as well as crystallinity are limited. For the purpose of stabilizing BOF slag, steel mills around the world have developed several treatment technologies [1,2,3,4,5,6]. Among these methods, hot slag modification [3,4,5,6] is a promising technology for BOF slag stabilization. To facilitate the utilization of BOF slag, China Steel Corporation (CSC) established a molten BOF slag modification station to convert liquid slag into stable BOF slag [5,6]. The modified BOF slag has properties of low expansion rate (<0.5%) and low pH (<12), which are helpful for BOF slag utilization. However, conventional molten BOF slag modification involves injecting 5–16% silica as an additive, which poses several challenges, including increased heat loss, higher processing costs, and an increase in slag volume [3,4,5,6].

In response to these challenges, this study introduces an innovative process for stabilizing BOF slag by blowing oxygen into the molten slag. Unlike traditional methods that rely on silica injections, this new process achieves stabilization solely through oxygen blowing. Compared to previous studies that stabilized BOF slag by remelting it in air [7,8]—an energy-intensive and industrially impractical approach—this study introduces a more efficient and scalable method. In addition, the required oxygen content for injection into conventional molten BOF slag to achieve stabilization has not been reported. In this work, molten BOF slag produced from our steelmaking workshop was directly modified at the slag modification station. The chemical compositions and crystalline phases were analyzed with varying amounts of oxygen injected during the modification process. In the industrial trials, blowing oxygen into the molten BOF slag facilitated the oxidation of FeO to Fe_2_O₃, which subsequently reacted with f-CaO to form volumetrically stable C_2_AF (brownmillerite, Ca_2_Al_x_Fe_2−x_O_5_). To understand the influence of oxygen blowing on the stabilization effect of BOF slag, 70 industrial trials were conducted (20–25 tons of slag per trial) with the basicity of the BOF slag ranging from 2.2 to 4.5. The results show that the expansion reduction is proportional to the oxygen-blowing amount. These results are consistent with the XRD and SEM-EDX data, which show that more f-CaO and RO phases are converted to the C_2_AF phase as the oxygen-blowing amount increases, thereby reducing the expansion rate of the slag samples.

The treated BOF slag exhibited excellent volumetric stability (expansion < 0.5%) and a lower pH (10.6–10.8) in comparison to original BOF slag. Additionally, leaching-test results confirmed its compliance with Taiwan’s EPA regulations. As a result, the modified BOF slag can be effectively utilized in various engineering applications, including pavement bricks, concrete products, and high-value-added engineered stones. Most importantly, since brownmillerite has shown potential applications in low-carbon cement [9], the increased presence of brownmillerite in BOF slag represents a promising advancement for the innovative and high-value utilization of BOF slag.

## 2. Materials and Methods

### 2.1. The Modification of Molten BOF Slag

**Figure 1:**
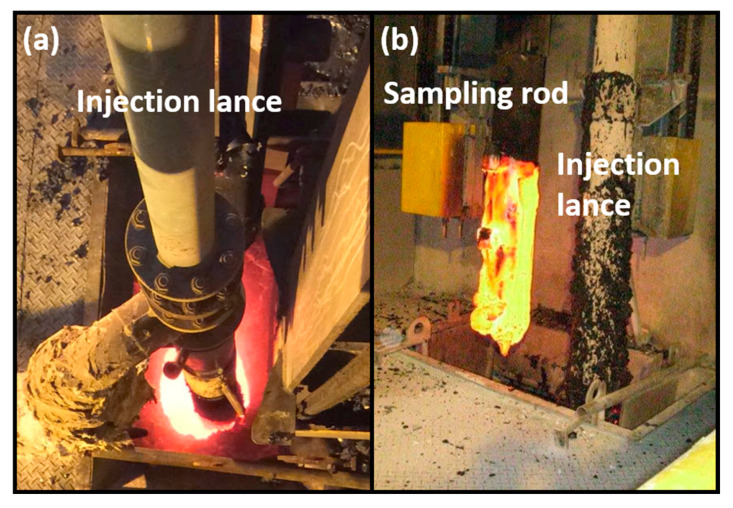
(**a**) Top view and (**b**) side view of molten BOF slag modification station during processing.

All experiments were conducted using the molten BOF slag modification station at CSC (Figure 1). Typically, a slag pot containing 20 tons of molten BOF slag (1346–1423 °C) was processed by injecting a mixture of oxygen and nitrogen into the slag through a lance. During the process, oxygen in the range of 60–180 Nm^3^ was blown into the molten slag. After the injection process was completed, molten slag samples (1365–1410 °C) were collected using a sampling rod (Figure 1b) for expansion rate measurements and further characterization.

### 2.2. Expansion Measurements of BOF Slag

In this study, volumetric stability refers to the volume changes in steel slag during hydration at ambient temperature. For instance, steel slag containing free lime tends to expand as free lime reacts with water at room temperature (CaO + H_2_O → Ca(OH)_2_). Thus, the expansion rate is primarily determined by the volumetric changes in slag components during hydration. To evaluate the expansion behavior of BOF slag, accelerated hydration tests and expansion measurements were conducted, and the stabilization effect was determined by comparing the slag’s expansion rate before and after modification. The slag powder was ground to into powder (<53 μm) using a planetary ball mill. Then, the powder was pressed into the stainless cylindrical (diameter and height = 1 cm) mold and compressed using a hydraulic press at a pressure of 7600 kgf/cm^2^ for 1 min. After compression, the height of the cylindrical pellet confined in the mold was measured using a caliper so that the initial volume (Vi) of the sample can be calculated by using 0.5 × 0.5 × π × Hi (initial height) with the unit of cm^3^. The pellet was then placed in a water bath at 90 °C for 30 min. Finally, the sample was taken out, and its height was measured again. Therefore, Vf can be calculated using 0.5 × 0.5 × π × Hf (final height) with the unit of cm^3^. Finally, the expansion rate was calculated using Equation (1). Successful modification is indicated when the expansion rate of the BOF slag after treatment falls below 0.4%.

Expansion rate = (Vf − Vi)/Vi × 100%(1)

Vi: the volume of BOF slag before modification;
Vf: the volume of BOF slag after modification.

### 2.3. XRD Measurements of BOF Slag

X-ray diffraction (XRD) measurements were conducted using a Malvern PANalytical EMPYREAN diffractometer (Malvern PANalytical, Almelo, The Netherlands) equipped with a Co radiation source (Kα1 = 1.7901 Å, Kα2 = 1.7929 Å), operated at 40.0 kV. The measurements were performed over a 2θ range of 10° to 83°, with a step size of 0.0131°. Quantitative phase analysis was carried out using the Rietveld refinement method with HighScore Plus software (version 4.9.0.27512), utilizing the PDF-4/Axiom 2023 database.

### 2.4. pH Measurements of BOF Slag

The pH value was determined in accordance with the Taiwanese EPA regulation (NIEA R208.04C). Slag particles smaller than 9.5 mm were collected and ground into powder (<1 mm). A 20 g sample of the slag powder was mixed with 20 mL of pure water and stirred for 5 min. The mixture was then left to settle for 15 min before filtration. Finally, the pH of the filtrate was measured using a pH electrode.

### 2.5. TCLP Tests of BOF Slag

TCLP (Toxicity Characteristic Leaching Procedure) tests were conducted in accordance with the regulations of Taiwan’s EPA. Samples were ground to a particle size smaller than 9.5 mm to ensure uniformity. A leaching solution with a pH of 2.88 ± 0.05, prepared using acetic acid, was used for the tests. The ground sample was mixed with the leaching solution at a solid-to-liquid ratio of 1:20. The mixture was placed in a rotary extraction device capable of rotating the extraction vessel (2.2 L) in an end-over-end fashion at 30 ± 2 rpm and agitated for 18 ± 2 h at room temperature (approximately 23 ± 2 °C) to simulate leaching conditions and ensure thorough contact between the solid waste and the solution. After agitation, the mixture was filtered to separate the liquid leachate from the solid residue. The leachate was analyzed for specific toxic contaminants, including heavy metals such as lead, mercury, and arsenic. The concentrations of these contaminants were measured using an Agilent 5800 inductively coupled plasma-optical emission spectrometer (ICP-OES; Agilent Technologies, Santa Clara, CA, USA).

## 3. Results and Discussion

### 3.1. Principle of BOF Slag Stabilization

**Figure 2:**
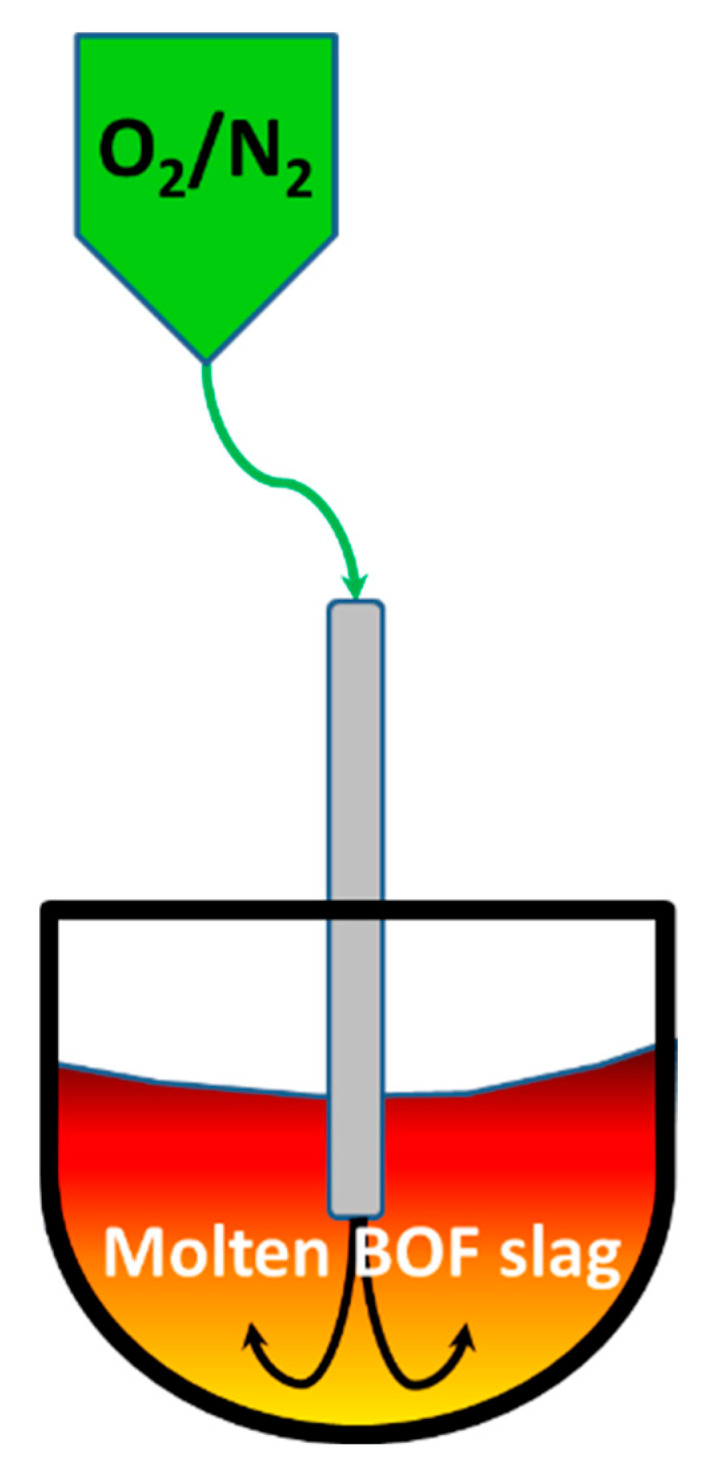
Schematic diagram of molten BOF slag modification through oxygen blowing.

Traditional strategies for stabilizing BOF slag involve reducing its basicity by injecting SiO_2_ into the molten slag [3,4,5,6]. The injected silica melts and reacts with the free lime (f-CaO) in the slag, forming volumetrically stable calcium silicates, as shown in Equation 2. However, depending on the basicity of the slag, an additional 5–16% of silica is required for injection. This approach leads to increased operating costs, larger slag volumes, and greater heat loss. To address these challenges, we developed a novel process for BOF slag stabilization. In the industrial trials, a mixture of oxygen and nitrogen is blown into the molten BOF slag without the addition of silica (Figure 2). Stabilization occurs through the oxidation of FeO in the slag, which forms Fe_2_O_3_. This Fe_2_O_3_ then reacts with f-CaO to produce stable brownmillerite [7,8], as shown in Equations (3) and (4). This process offers several advantages, including reduced operating costs, less slag volume produced, and minimized heat loss, representing a significant improvement over traditional methods.

2CaO(free) + SiO_2_ → Ca_2_SiO_4_ (C_2_S) (2)

2FeO + 0.5O_2_ → Fe_2_O_3_ (3)

2CaO(free) + 0.5x Al_2_O_3_ + 0.5(1 − x) Fe_2_O_3_ → Ca_2_Al_x_,Fe_2−x_O_5_ (C_2_AF) (4)

### 3.2. Mechanistic Study of BOF Slag Stabilization by Oxygen Blowing

**Figure 3:**
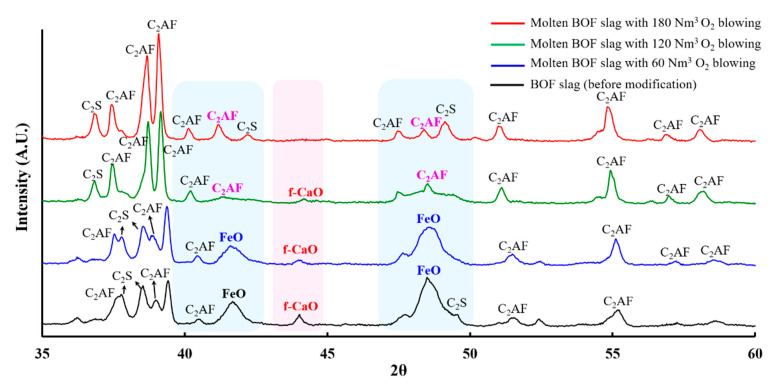
XRD patterns of BOF slag treated with oxygen blowing at 0, 60, 120, and 180 Nm^3^, respectively.

To investigate the effect of oxygen blowing on BOF slag stabilization, molten slag samples treated by 0, 60, 120, and 180 Nm^3^ of oxygen blowing were collected, slowly cooled by air, and analyzed using XRD and SEM-EDX techniques. Due to the slow air cooling, all slag samples exhibited well-developed crystalline phases, and the glassy phase content remained unchanged throughout the transformation process. The XRD analysis (Figure 3) revealed a gradual decrease in free lime (f-CaO) as the oxygen-blowing volume increased from 0 to 120 Nm^3^, with complete disappearance after blowing 180 Nm^3^ of oxygen. Additionally, the f-CaO peak shifts toward higher 2θ values during the transformation, indicating the gradual incorporation of FeO and MgO into the lime phase [10]. Similarly, the content of the RO phase (a solid solution of FeO, MgO, and MnO) decreases with increasing oxygen blowing, suggesting that the RO phase begins to react with f-CaO to form brownmillerite. Quantitative analysis (Table 1) shows that the RO phase decreased from 34.1% to 3.8%, and f-CaO reduced from 2.9% to 0%. Meanwhile, the C_2_AF (brownmillerite) phase significantly increased from 21.3% to 61.1% as the oxygen-blowing volume rose from 0 to 180 Nm^3^. This suggests that oxygen blowing promotes the conversion of f-CaO and FeO into the C_2_AF phase, aligning with previous studies by FactSage, with calculations indicating that the C_2_AF phase is the dominant phase when molten slag is exposed to air [7,8]. Additionally, XRD patterns (Figure 3) show a shift in the C_2_AF peaks toward lower 2θ values during the transformation, suggesting that the newly formed brownmillerite gradually transitions to a srebrodolskite (Ca_2_Fe_2_O_5_)-like phase. The sharpening of these peaks with increased oxygen blowing further implies improved crystal size and crystallinity.

**Figure 4:**
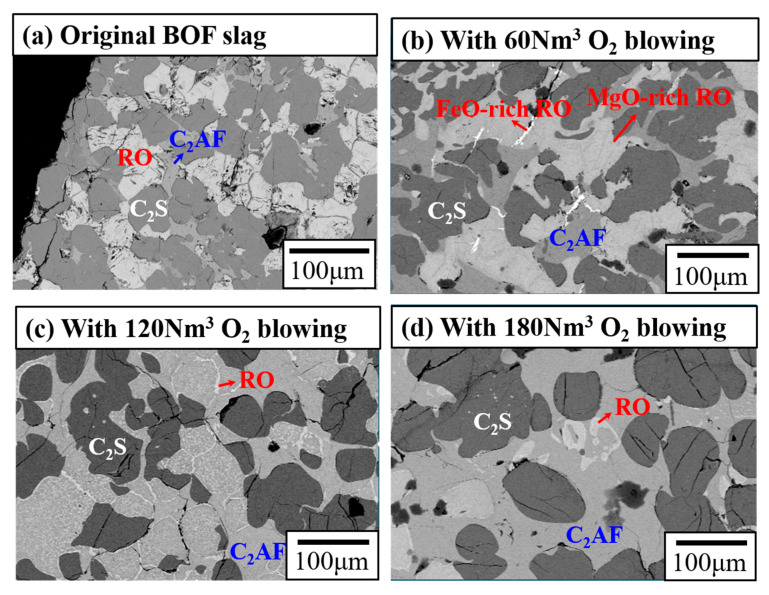
SEM images of molten BOF slag blown by (**a**) 0 Nm^3^, (**b**) 60 Nm^3^, (**c**) 120 Nm^3^, and (**d**) 180 Nm^3^ of oxygen.

Using the SEM-EDX analysis (Figure 4), the distribution and transitions of the C_2_S, RO, and C_2_AF phases were monitored. In Figure 4a, C_2_S and RO are shown as the predominant phases, while C_2_AF appears as a minor phase. With 60 Nm^3^ of oxygen blowing, the RO phase is observed to separate into two distinct domains. As illustrated in Figure 4b, the major domain is MgO-rich RO, while the minor domain (bright areas in Figure 4b) represents FeO-rich RO. This result suggests that FeO in the RO phase is initially oxidized and subsequently reacts with f-CaO to form C_2_AF. When 120 Nm^3^ of oxygen is blown, the RO phase decreases significantly, and the C_2_AF phase increases substantially (Figure 4c). Finally, with 180 Nm^3^ of oxygen blowing, the C_2_AF phase becomes predominant, leaving only traces of the RO phase (Figure 4d). By tracking the Ca/Fe ratio within the C_2_AF phase, it was found that the ratio progressively declined from 1.9 in the original BOF slag to 1.2 after applying 180 Nm^3^ of oxygen blowing. This decrease reflects the progressive oxidation of FeO to Fe_2_O₃, leading to greater Fe^3^⁺ substitution for Al^3^⁺ in the C_2_AF structure (Ca_2_Al_x_Fe_2_₋_x_O_5_). As a result, the C_2_AF phase evolves toward a srebrodolskite-like composition (Ca_2_Fe_2_O_5_). With the majority of iron oxidized to Fe^3^⁺ within C_2_AF, the modified slag is anticipated to demonstrate excellent phosphorus extraction selectivity, enhancing its value as a resource [11]. Moreover, the transformation into a srebrodolskite-like phase suggests a reduced chromium-leaching risk, as srebrodolskite exhibits lower solubility [12,13]. These observations are consistent with the XRD analysis (Figure 3), which shows that the RO phase diminishes as oxygen blowing increases, while the C_2_AF phase becomes increasingly dominant, progressively evolving into a continuous srebrodolskite (Ca_2_Fe_2_O_5_)-like phase (Figure 4d). Additionally, the crystal sizes of the C_2_S phase are observed to grow from 30–50 µm to 50–100 µm (Figure 4d) as the oxygen volume increases from 0 to 180 Nm^3^, which is in agreement with the XRD’s findings. These results confirm that BOF slag stabilization occurs via the reactions outlined in Equations (3) and (4). They also highlight the critical role of the oxygen-blowing volume in achieving effective stabilization, transforming f-CaO and FeO into stable phases with improved crystallinity.

### 3.3. Conditions for Stabilization of BOF Slag by Oxygen Blowing

**Figure 5:**
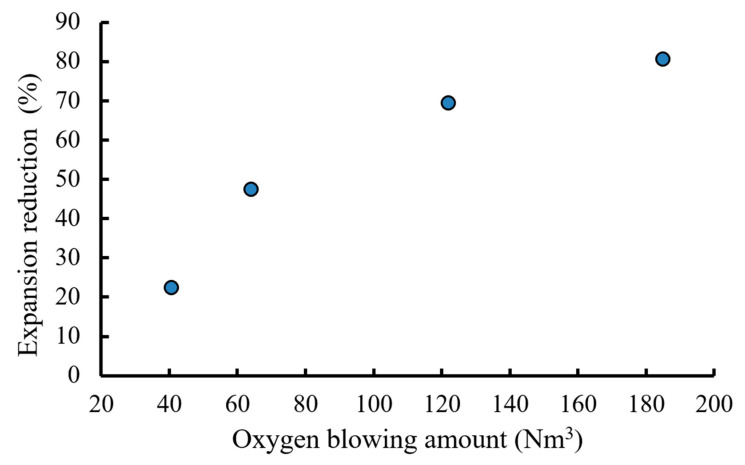
Expansion reduction in BOF slag samples under varying oxygen-blowing amounts.

To understand the influence of oxygen blowing on the stabilization effect of BOF slag, 70 industrial trials were conducted (20–25 tons of slag per trial) with the basicity of the BOF slag ranging from 2.2 to 4.5 (Table S1 in the Supplementary Materials). Since different BOF slag samples exhibited varying expansion values, the expansion reduction was calculated according to Equation (5) for each sample before and after treatment. Consequently, the stabilization effect of different oxygen-blowing amounts could be evaluated. The results presented in Figure 5 reveal that the expansion reduction is proportional to the oxygen-blowing amount. Specifically, the expansion reduction was only 22.5% when 40.6 Nm^3^ of oxygen was blown. As the oxygen-blowing amount increased, the expansion reduction also rose, ultimately reaching 80.7% at 184.5 Nm^3^ of oxygen. These results are consistent with the XRD and SEM-EDX data, which show that more f-CaO and RO phases are converted to the C_2_AF phase as the oxygen-blowing amount increases, thereby reducing the expansion rate of the slag samples. To understand the influence of basicity on the expansion reduction, we analyzed the expansion reductions in BOF slags with different basicities when the oxygen-blowing amount is larger than 180 Nm^3^. Finally, it was found that BOF slag with higher basicity (CaO/SiO_2_ > 4) achieved a significantly greater expansion reduction (91.9%) compared to slag with lower basicity (CaO/SiO_2_ < 4), which showed an average reduction of 66.7%. These results indicate that our approach is effective for stabilizing high-basicity BOF slag, which typically exhibits a high expansion rate.

Expansion reduction (%) = (Ei − Ef)/Ei × 100%(5)

Ei: the expansion value of BOF slag before treatment
Ef: the expansion value of BOF slag after treatment

### 3.4. Properties of Modified BOF Slag and Its Applications

**Figure 6:**
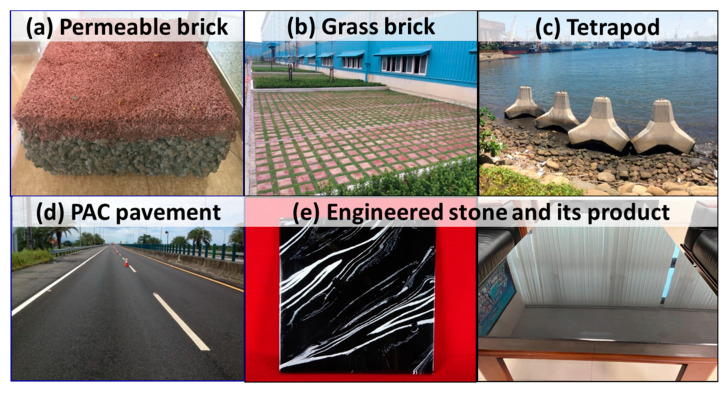
Aggregates of modified BOF slag applied in (**a**) permeable brick, (**b**) grass brick, (**c**) tetrapod, (**d**) porous AC (PAC) pavement, and (**e**) high-value-added engineered stone.

To explore potential applications of modified BOF slag, a detailed characterization of its properties was conducted. Table 2 presents a comparison of the crystalline phases and characteristics of BOF slag before and after oxygen blowing. Without oxygen blowing, the BOF slag predominantly contains 42% C_2_S (2CaO·SiO_2_), 21% C_2_AF (Ca_2_Al_x_Fe_2_₋_x_O_5_), 34% RO (FeO/MgO/MnO), and 3% free lime (f-CaO). In contrast, after oxygen blowing, significant changes occur in the crystal composition. The C_2_AF phase increases substantially to 61%, while the C_2_S phase decreases to 35%. The RO phase is notably reduced to just 4%, and f-CaO is completely eliminated. Despite the modification, the basicity (B_2_, CaO/SiO_2_) remains within the same range of 3.8–4.7. However, the expansion rate decreases drastically from 3–6% in untreated BOF slag to below 0.5% in oxygen-blown slag. Additionally, the pH value decreases from 12.4 to 10.8, indicating reduced alkalinity in the modified slag due to the absence of f-CaO. As shown in the XRD patterns (Figure 3), the newly formed brownmillerite gradually transitions into a srebrodolskite (Ca_2_Fe_2_O_5_)-like phase with increasing oxygen blowing. This suggests that the leaching risk of chromium is reduced, as srebrodolskite exhibits lower solubility [12,13]. To ensure environmental safety, TCLP (Toxicity Characteristic Leaching Procedure) tests were performed to evaluate the leaching properties of the modified BOF slag. The results presented in Table 3 confirm that the leaching properties of the modified BOF slag meet Taiwan’s EPA regulations. These findings confirm that this approach effectively improves the volumetric stability of the slag while reducing its expansion rate, alkalinity, and leaching risks. Also, in comparison to natural aggregates, superior soundness property of the modified BOF slag is demonstrated. Instead of soundness loss of 3 in natural aggregates, nearly no soundness loss is revealed in the modified BOF slag. This result indicates that the modified BOF slag has excellent durability. These improvements make the modified BOF slag highly suitable for making pavement/grass bricks (Figure 6a,b), tetrapod concrete products (Figure 6c) [5], PAC (Porous Asphalt Concrete) road (Figure 6d) [14], and engineering stone (Figure 6e) [15], respectively. For pavement/grass bricks, modified BOF slag was crushed to aggregates with size of 1 cm, which were then mixed with 20% cement to fabricate bricks. It was found that the compressive strength of the modified BOF-slag-based bricks is generally higher than 70 MPa, which shows a better mechanical property than the ones with substitution by nature aggregates. Also, tetrapod concrete product (Figure 6c, 10 t/unit) was produced in similar manner. Normally aggregates of modified BOF slag were mixed with sands and cement to produce the concrete. The concrete product exhibits excellent volumetric stability in outdoor tests. In addition to the aforementioned applications, modified BOF slag was utilized for substituting natural aggregates in PAC roads (Figure 6d). In the preliminary examination, good qualities of these modified BOF-slag-based products were shown. Besides conventional uses of modified BOF slag, the development of high-value-added products, such as engineered stone (Figure 6e), is in progress. Since this approach generates a high amount of C_2_AF (~60%), it exhibits strong potential for low-carbon cement applications [9,16]. Furthermore, with the majority of iron oxidized to Fe^3^⁺, the modified slag is expected to exhibit excellent phosphorus extraction selectivity, enhancing its potential as a valuable resource [11]. Overall, ongoing research aims to further characterize the modified BOF slag and explore innovative applications for this versatile material [17,18].

## 4. Conclusions

In this study, a novel process for BOF slag stabilization was developed and successfully implemented at the slag modification station of CSC. Unlike conventional methods that involve injecting silica into molten BOF slag, this innovative approach requires only the blowing of an oxygen–nitrogen mixture. Stabilization is achieved through the oxidation of FeO in the slag, forming Fe_2_O_3_, which subsequently reacts with free lime (f-CaO) to produce stable brownmillerite. The use of an oxygen–nitrogen mixture in this process offers significant advantages, including lower operating costs, less slag produced, and minimized heat loss. The modified BOF slag exhibits superior characteristics, such as minimal expansion (<0.5%), a lower pH (10.6–10.8) in comparison to original BOF slag, and the absence of toxic material leaching. These properties make it highly suitable for various applications, including road pavements, concrete products, and high-value engineering materials. Additionally, the high calcium ferrite content in the modified slag underscores its potential as a cementitious material for low-carbon cement applications and other functional materials.

## Figures and Tables

**Table 1:** Quantitative analysis of crystalline phases in BOF slag under varying oxygen-blowing amounts.

	Original BOF Slag	60 Nm^3^ O_2_ Blowing	120 Nm^3^ O_2_ Blowing	180 Nm^3^ O_2_ Blowing
C_2_AF (Ca_2_Al_x_Fe_2−x_O_5_)	21.3%	29.8%	44.9%	61.1%
C_2_S (2CaO·SiO_2_)	41.7%	36.8%	34.7%	35.1%
RO (FeO/MgO/MnO)	34.1%	32.9%	21.2%	3.8%
f-CaO	2.9%	0.5%	0.2%	0%

**Table 2:** Comparison of crystalline phases in BOF slag with and without oxygen blowing.

Characteristics		BOF Slag Without Oxygen Blowing	BOF Slag with Oxygen Blowing
Crystal phase	C_2_S (2CaO·SiO_2_)	42%	35%
	C_2_AF (Ca_2_Al_x_Fe_2−x_O_5_)	21%	61%
	RO (FeO/MgO/MnO)	34%	4%
	f-CaO	3%	0%
B_2_ (CaO/SiO_2_)		3.8–4.7	3.8–4.7
Expansion rate (%)		3–6	<0.5
pH		12.4	10.8

**Table 3:** TCLP test results of BOF slag with oxygen blowing at 60, 120, and 180 Nm^3^, respectively.

Elements	Methods	Standard (mg/L)	Modified BOF Slag (60 Nm^3^ O_2_ Blowing) (mg/L)	Modified BOF Slag (120 Nm^3^ O_2_ Blowing) (mg/L)	Modified BOF Slag (180 Nm^3^ O_2_ Blowing) (mg/L)
Pb	NIEA R317.11C	<5.0	N.D.	N.D.	N.D.
Cd	NIEA R317.11C	<1.0	N.D.	N.D.	N.D.
Cu	NIEA R317.11C	<15.0	N.D.	N.D.	N.D.
Cr	NIEA R317.11C	<5.0	<0.111	<0.111	<0.111
As	NIEA R317.11C	<5.0	N.D.	N.D.	N.D.
Hg	NIEA R317.11C	<0.2	N.D.	N.D.	N.D.
Se	NIEA R317.11C	<1.0	N.D.	N.D.	N.D.
Cr^6+^	UV analysis	<2.5	<0.01	<0.01	<0.01

Note: N.D. = Not detected, below method detection limit.

## Data Availability

The original contributions presented in this study are included in the article/Supplementary Materials. Further inquiries can be directed to the corresponding author.

## Supplementary Materials

The following supporting information can be downloaded at: https://www.mdpi.com/article/10.3390/ma18102182/s1, Table S1. 70 Industrial raw data with different basicity, O_2_ blowing amount (Nm^3^) and expansion reduction (%) are listed.
