# Supplementary material for: Industrial-Scale Brownmillerite Formation in Oxygen-Blown Basic Oxygen Furnace Slag: A Novel Stabilization Approach for Sustainable Utilization
Source: Materials (Basel). 2025 May 9;18(10):2182. doi: 10.3390/ma18102182 (PMC12112785; doi:10.3390/ma18102182)
Supplement: Supplementary file 1 [file materials-18-02182-s001.zip › materials-3556775-supplementary.pdf]

Supplementary Materials

Industrial-Scale Brownmillerite Formation in Oxygen-Blown Basic Oxygen Furnace Slag: A Novel Stabilization Approach for Sustainable Utilization

Yao-Hung Tseng[1,*], Yu-Hsien Kuo [2] and Meng-Hsun Tsai [2]

[1] New Materials Research and Development Department, China Steel Corporation, Taiwan; yaohung.tseng@mail.csc.com.tw
[2] Intellectual Property and Testing Technology Department, China Steel Corporation, Taiwan; 179259@mail.csc.com.tw; 204362@mai.csc.com.tw
* Correspondence: yaohung.tseng@gmail.com

Table S1. 70 Industrial raw data with different basicity, O_2 blowing amount (Nm^3) and expansion reduction (%) are listed.

Table S1

CaO/SiO_2	O_2 (Nm^3)	Expansion reduction (%)
4.3	34.1	31.0%
4.18	41.62	-1.1%
4.3	41.62	27.7%
3.665	41.7	37.8%
3.405	42.32	2.0%
3.86	42.48	37.5%
3.76	56.13	57.7%
4.1	62.37	97.6%
3.1	63.96	48.9%
3.615	63.98	89.6%
4.455	64.08	39.3%
2.63	64.15	-16.6%
3.91	64.75	48.5%
3.87	65.07	85.5%
4.28	65.15	28.2%
3.44	65.17	18.4%
4.02	68.84	24.4%
2.23	69.19	48.6%

CaO/SiO_2	O_2 (Nm^3)	Expansion reduction (%)
4.43	78.04	79.8%
4.3	78.24	89.4%
4.195	79.05	93.3%
3.7	79.34	98.1%
4.205	80.1	82.7%
4.41	80.68	63.6%
3.74	80.75	7.9%
4.34	80.77	95.1%
3.89	80.78	89.2%
3.88	81.16	93.2%
4.235	82.38	95.2%
3.62	83.44	44.8%
4.485	83.45	44.8%
3.99	83.48	89.5%
4.265	102.67	9.7%
3.935	118.45	85.9%
3.33	118.5	66.8%
3.02	118.53	55.2%
3.45	118.62	-1.4%
3.96	118.67	92.4%
3.435	118.69	95.2%
3.33	119.76	30.6%
3.785	119.76	81.3%
4.4	119.77	50.9%
3.71	119.8	90.2%
4.5	119.89	99.8%
3.495	121.51	98.4%
4.295	122.86	41.3%
4.44	123.13	51.0%
4.4	124.02	21.1%
3.6	125.63	85.7%
4.265	125.71	94.1%
3.18	125.75	94.0%
4.34	125.84	97.6%
4.35	127.82	90.8%

CaO/SiO_2	O_2 (Nm^3)	Expansion reduction (%)
4.36	142.94	89.1%
3.53	143.06	98.6%
3.575	143.06	83.3%
4.04	143.1	95.8%
3.66	143.12	98.0%
4.36	159.04	97.0%
4.16	161.76	99.9%
4.245	182.94	65.4%
4.4	183.58	99.6%
3.83	184.03	99.4%
3.1	184.26	51.7%
3.995	184.46	27.1%
4.175	184.72	99.3%
4.22	185.24	95.7%
4	185.45	99.7%
3.37	185.89	88.4%
